# Disrupted functional connectivity between visual and emotional networks in psychosis risk syndromes through representational similarity analysis

**DOI:** 10.3389/fpsyt.2025.1533675

**Published:** 2025-04-17

**Authors:** Xi Ruan, Lang Zhang, Mingjun Duan, Dezhong Yao, Cheng Luo, Hui He

**Affiliations:** ^1^ The Clinical Hospital of Chengdu Brain Science Institute, MOE Key Lab for Neuroinformation, University of Electronic Science and Technology of China, Chengdu, China; ^2^ High-Field Magnetic Resonance Brain Imaging Key Laboratory of Sichuan Province, School of Life Science and Technology, University of Electronic Science and Technology of China, Chengdu, China; ^3^ Research Unit of NeuroInformation, Chinese Academy of Medical Sciences, Chengdu, China

**Keywords:** psychosis risk syndromes, fMRI, representational similarity analysis, visual network psychosis risk syndromes, visual network

## Abstract

Schizophrenic individuals experience a prolonged prodrome before their first episode, often referred to as Psychosis Risk Syndromes (PRS). The PRS is characterized by non-specific symptoms, yet the underlying neural mechanisms remain unclear. Representational similarity analysis (RSA) has proven effective in elucidating the relationships between different data modalities. This approach could provide valuable insights into the functional coupling between sensory perception and emotion in PRS subjects. In this study, there were 27 PRS subjects and 33 control subjects. Neuropsychological assessments were conducted to evaluate the participants’ recent mental states and their risk of mental illness. Each subject underwent task-based functional magnetic resonance imaging (fMRI), which included steady-state visual evoked potentials (SSVEP) and expression matching tasks. The areas of brain activity were defined as regions of interest (ROIs). RSA was used to calculate the relationships between the SSVEP and expression matching tasks. In the functional coupling between the SSVEP at 5 Hz and 10 Hz conditions, the PRS group showed lower functional coupling in the fusiform area compared to controls. Additionally, in the functional coupling between the SSVEP at 10 Hz and the emotion matching conditions, the PRS group demonstrated decreased activation in visual regions compared to controls. Overall, our findings suggest that PRS subjects exhibit diminished functional couplings between basic visual stimuli and vision-emotion matching tasks, indicating abnormal visual processing in both the primary visual cortex and more advanced stages of information processing.

## 1 Introduction

Schizophrenia is a complex mental disorder, and its symptoms are typically divided into positive, negative, and cognitive types (1, 2). Patients often exhibit deficits in cognition, emotion, and perception, with abnormal visual processing being a notable impairment (3). Landgraf posits that vision may play a central role in the onset and progression of schizophrenia (4). Studies have shown that the positive symptoms of schizophrenia are associated with selective attention deficits within the visual system (5). These deficits impede patients’ ability to extract relevant information from complex environments and filter out irrelevant visual stimuli, hindering their capacity to integrate necessary information at a whole-brain level (5, 6). Previous research has identified deficits in visual processing across various stimulus types, such as graphic face recognition (7) and drawing (8), suggesting an impairment of the visual network in individuals with schizophrenia. It has also been found that clinically high-risk groups (Psychosis Risk Syndromes, or PRS) that eventually convert to schizophrenia perform significantly worse on visual memory and visuospatial working memory tasks compared to those who do not convert (9). A meta-analysis by Fusar-Poli et al. confirms that the transition to psychosis is characterized by impairments in visual memory and working memory (10). Given that schizophrenia has a long latency period from initial onset to diagnosis, this timeframe is referred to as the high-risk period or clinical high-risk period. Research in this period offers valuable insights for understanding the development of schizophrenia and other serious mental disorders. Visual abnormalities are a core symptom of schizophrenia; thus, studying the visual processing of psychiatric risk groups is crucial for exploring the progression of these diseases.

Initially, SSVEP was primarily utilized in electroencephalogram (EEG) research, where EEG technology was used to collect and analyze signals from the visual cortex of the brain to explore firing patterns. Evoked potentials can arise in two ways: they can be induced by physical sensory stimulation, referred to as exogenous induction, or by internal cognition and movement, known as endogenous induction. SSVEP refers to an exogenous evoked potential generated by visually related fixed-frequency physical stimulation in the visual cortex (11). Previous studies have indicated that due to the adaptability of the visual system, prolonged exposure to a stimulus results in the visual system’s response becoming increasingly stable. Additionally, SSVEP results are generally more reliable and less affected by artifacts caused by physiological reactions such as blinking or eye movements, and they exhibit excellent anti-noise performance (12). There are various methods to generate SSVEP, with commonly used stimuli including flickering lights or images, and flickering Gaussian noise (13). The human brain consists of numerous regions with distinct functions, and different functional regions respond differently to stimuli of varying forms or frequencies (14). Therefore, selecting the appropriate research frequency must be targeted and based on the research content. Herrmann (15) found that humans achieve peak visual resonance at 10 Hz, 20 Hz, 40 Hz, and 80 Hz with non-patterned visual flicker stimuli (15). Furthermore, the current SSVEP stimulation frequency used in brain-computer interfaces typically falls within the low-frequency band, as low-spatial frequency stimulation contains more global information. In this experiment, we selected the three stimulation frequency bands of 5 Hz, 10 Hz, and 20 Hz.

To explore the relationship between the visual network in the psychiatric risk group and other networks, we also investigated the emotional face matching experiment. Social cognition is a psychological process through which we perceive, encode, store, retrieve, and regulate facial information about others and ourselves to facilitate social interactions (16). It plays a crucial role in processing facial information. As a key component of social cognition, emotion recognition is essential in our daily lives; recognizing emotional faces impacts our interpersonal relationships, which, in turn, affects our overall quality of life. Studies investigating emotional face recognition in patients with schizophrenia have found that both facial recognition and facial emotion processing are impaired in these individuals (17).

Over the past decade, methods of representational similarity analysis (RSA) have become a popular approach for investigating patterns of brain activity (18). With advancements in technology, RSA has been utilized in functional neuroimaging to explore topics such as object classification, semantics, object recognition, emotion, action observation, and fear conditioning. Currently, this technique has been applied in psychiatry to examine the differences in representational spaces between individuals with mental disorders and healthy individuals (19). In addition to characterizing localized neural representations, RSA has proven particularly valuable for assessing functional coupling across distributed brain regions. By quantifying the similarity of activity patterns across different voxels and tasks, RSA enables a deeper understanding of the dynamic interplay and connectivity underlying complex brain functions.

## 2 Methods and materials

### 2.1 Subjects selection

Subjects were come from the University of Electronic Science and Technology of China. Symptom Check List 90 and Prodromal Questionnaire (PQ-16) were used to assess the psychosis-risk of college students. When a subject’s SCL-90 score is greater than 160 and their PQ-16 score is greater than 6, the subject is classified into the high-risk group. On the other hand, subject was classified into low-risk group (SCL-90 score < 160, PQ-16<6). Additionally, we excluded individuals with significant comorbid psychiatric conditions or those currently using psychotropic medications to minimize confounding factors. All procedures were approved by the Ethics Committee of the Clinical Hospital of Chengdu Brain Science Institute in accordance with the Helsinki Declaration. Each subject gave written, informed consent acknowledging before the beginning of the study. Subjects were free to withdraw from this study at any time.

### 2.2 Study design

In this experiment, alternating black and white images were used to simulate the steady-state visual evoked potential (SSVEP). The frequency stimuli included 5 Hz, 10 Hz, and 20 Hz. Before the experiment began, the participants were informed about the content and duration of the study. During the experiment, they were instructed to focus on the screen, avoid blinking, clear their minds, and keep their heads still. Throughout the task, we monitored the participants’ head movement; if their movements exceeded a predefined range, we would pause the session and collect data again. After each collection, we reminded the participants to continue staring at the screen and to take the experiment seriously. The total duration of the SSVEP task was 5 minutes and 50 seconds, which included 10 seconds of preparation and 340 seconds of task and rest periods. To avoid functional coupling effects and improve data quality, the task and rest periods were alternated. The entire session consisted of 17 blocks, each lasting 20 seconds, with task stimuli for each frequency presented three times, resulting in nine blocks of task stimuli and eight blocks of rest.

The emotional face matching experiment primarily investigates the activation of emotion-related brain regions and the process of brain recognition of emotions. A total of 30 different emotional images were used, including 15 depicting anger and 15 depicting fear, along with 4 neutral images. Before the experiment began, we explained the example image and the key response procedure. The emotional images were designed so that the target emotion served as a reference, and participants selected a key based on this target. Each emotional image featured three expressions arranged in a triangle, with the target expression at the apex and the other two at the corners. Participants chose which of the two expressions corresponded to the target. If the left expression matched the target, they pressed button 1; if the right one matched, they pressed button 3. The neutral stimuli were treated similarly, but the expressions were represented as oval shapes.

**Figure 1:**
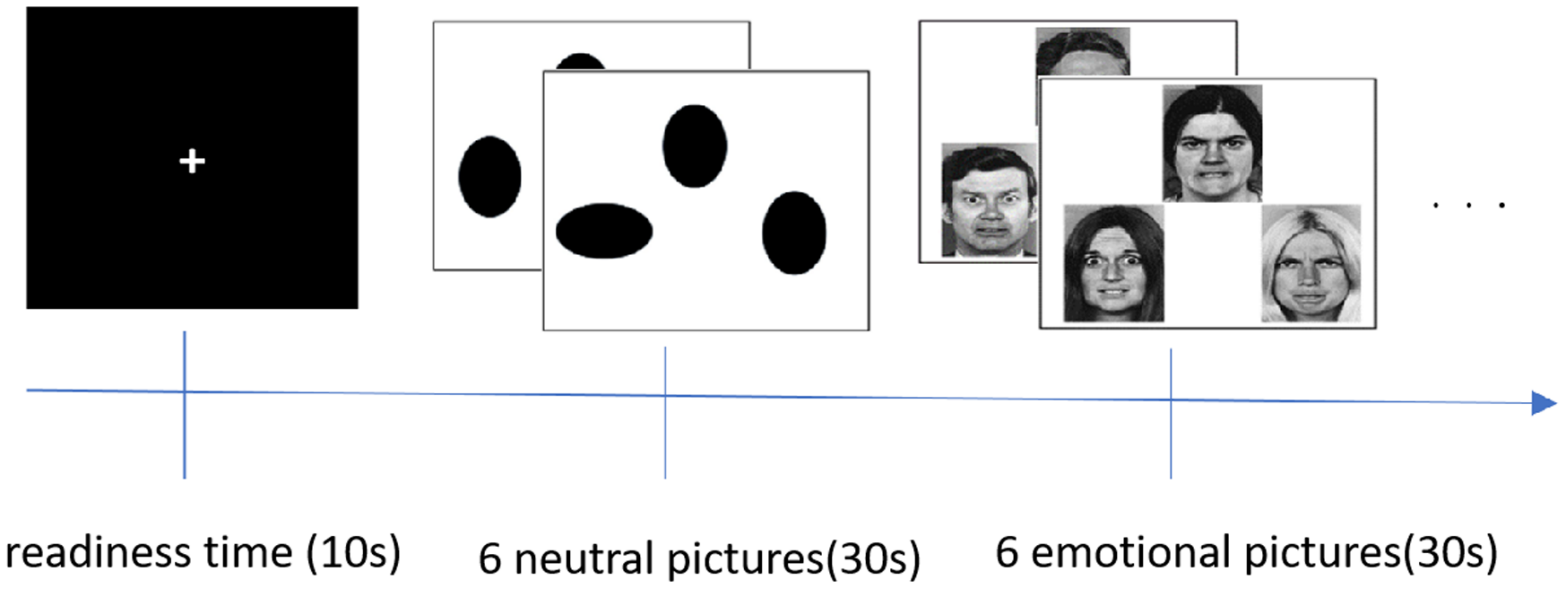
The design of emotional pictures stimulus.

The emotional face matching task lasted 5 minutes and 10 seconds, which included a 10-second preparation period at the start of the scan. Each block lasted 30 seconds and consisted of 10 blocks of task stimuli, comprising 5 blocks each of emotional and neutral stimuli. Emotional and neutral stimuli were presented alternately throughout the experiment. Each emotional stimulus block included 6 emotional pictures, and all 30 emotional pictures were displayed once during the entire session. The 4 types of neutral images appeared 30 times alternately throughout the experiment. To accurately record whether participants’ key press responses were correct, the expression stimuli were arranged in a specific order rather than randomly. The detailed experimental design and schematic diagram of the experiment are shown in **Figure 1**.

### 2.3 Imaging acquisition

Imaging was conducted on a 3T MRI scanner (GE DISCOVERY MR750). During scanning, we used foam padding and ear plugs to reduce head motion and scanner noise, respectively. Each subject underwent task stimulation in SSVEP and emotional face conditions during fMRI scan respectively. The fMRI data were acquired using gradient-echo echo planar imaging sequences (TR = 2000 ms, TE = 30 ms, FA= 90°, matrix = 64 × 64, FOV = 240 × 240 mm^2^, slice thickness/gap = 4 mm/0.4 mm, number of slices = 39), with an eight channel-phased array head coil. All subjects underwent a 175 volumes in SSVEP condition and 155 volumes in emotional face stimulation. During fMRI, all subjects were instructed to have their eyes-closed and to move as little as possible.

### 2.4 fMRI preprocessing

Functional imaging data were preprocessed using SPM12 according to a standard pipeline and only briefly described here. Slice time correction, head motion correction, normalization (3mm × 3mm × 3mm) into EPI template, and image smoothing (FWHM 6 mm) were carried out. Any subject who had a maximum translation in any of the orthogonal directions larger than 1.5 mm or rotation larger than 1.5 degree were excluded from subsequent analysis.

### 2.5 Task-based functional activity analysis

We conduct functional activation analysis. the first step is to conduct individual level analysis, all of which we carry out using the SPM toolkit. In order to further explore whether there are differences between the two groups of data, we also conducted a 2*3 repeated measures ANOVA on the two groups of data, and the results showed that the main effect and interaction effect between the two groups were not significant in either the SSVEP experiment or the emotional face matching task.

### 2.6 RSA analysis

**Figure 2:**
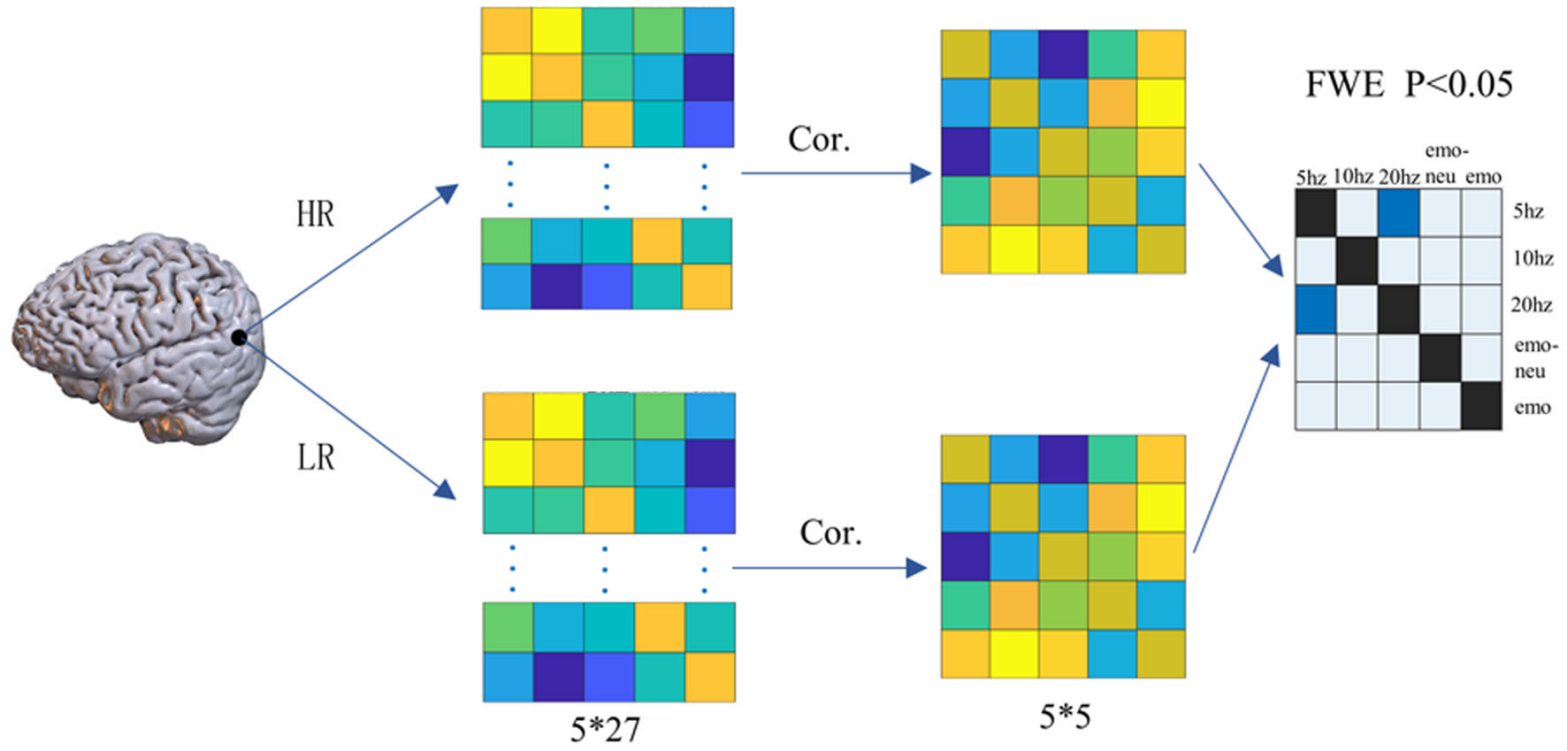
The steps of RSA analysis.

We used RSA analysis to explore the coupling between the same voxel in the SSVEP task and the emotional face matching experiment. The specific steps are as follows: (1) To determine the region of interest, we calculated the single-sample activation value of each condition in each task state, and determined our region of interest by the value of the one-sample t-test of the two groups of subjects. We used a total of five single-sample results to extract the coordinates of the region of interest using the three experimental conditions of the SSVEP and the two conditions of the expression face matching experiment. We defined regions of interest (ROI) based on the single-sample results under the five conditions, and then we took the coordinates of ROI as the center point and the radius of 6mm to extract the activation values in the high-risk group and the low-risk group under each condition. Finally, 5*27 matrices were obtained for each ROI of each subject, and then Pearson correlation between each column of the matrix and the other columns was calculated to obtain a 5*5 correlation matrix. Finally, two-sample T test was used to calculate and verify the similarity matrix of the two groups of subjects (FWE p<0.05). The detailed calculation process of RSA is shown in **Figure 2**.

## 3 Results

### 3.1 Demographics and characteristics

Data of a total of 60 subjects were used in this study, including 34 in the high-risk group and 38in the low-risk group. There was no significant difference in gender or age between the two groups of subjects (**Table1**).

**Table 1:** The demographics of two groups.

	High risk group M(SD)	Low risk group M(SD)	p
Gender (male/female)	25/9	29/9	0.785^a^
Age (year)	18.35 (0.13)	18.55 (0.14)	0.316^b^

^a^indicates the p values for the comparisons (Chi-square test) between two groups.

^b^indicates the p values for the comparisons (Two-sample t-tests) between two groups.

### 3.2 The results of functional activity

**Figure 3:**
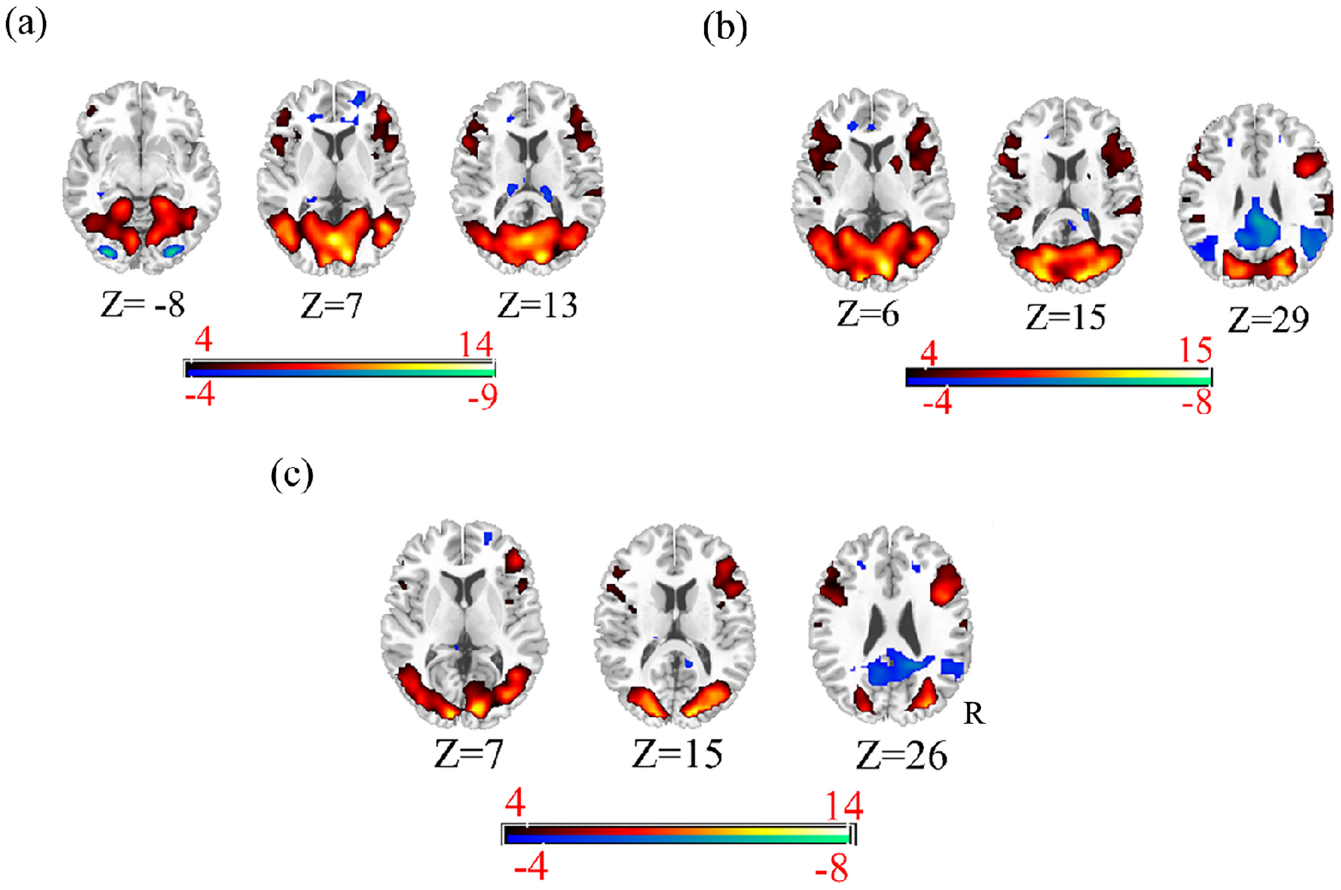
The functional activity mappings of ssvep: **(a)** 5Hz condition **(b)** 10Hz condition **(c)** 20Hz condition.

**Figure 4:**
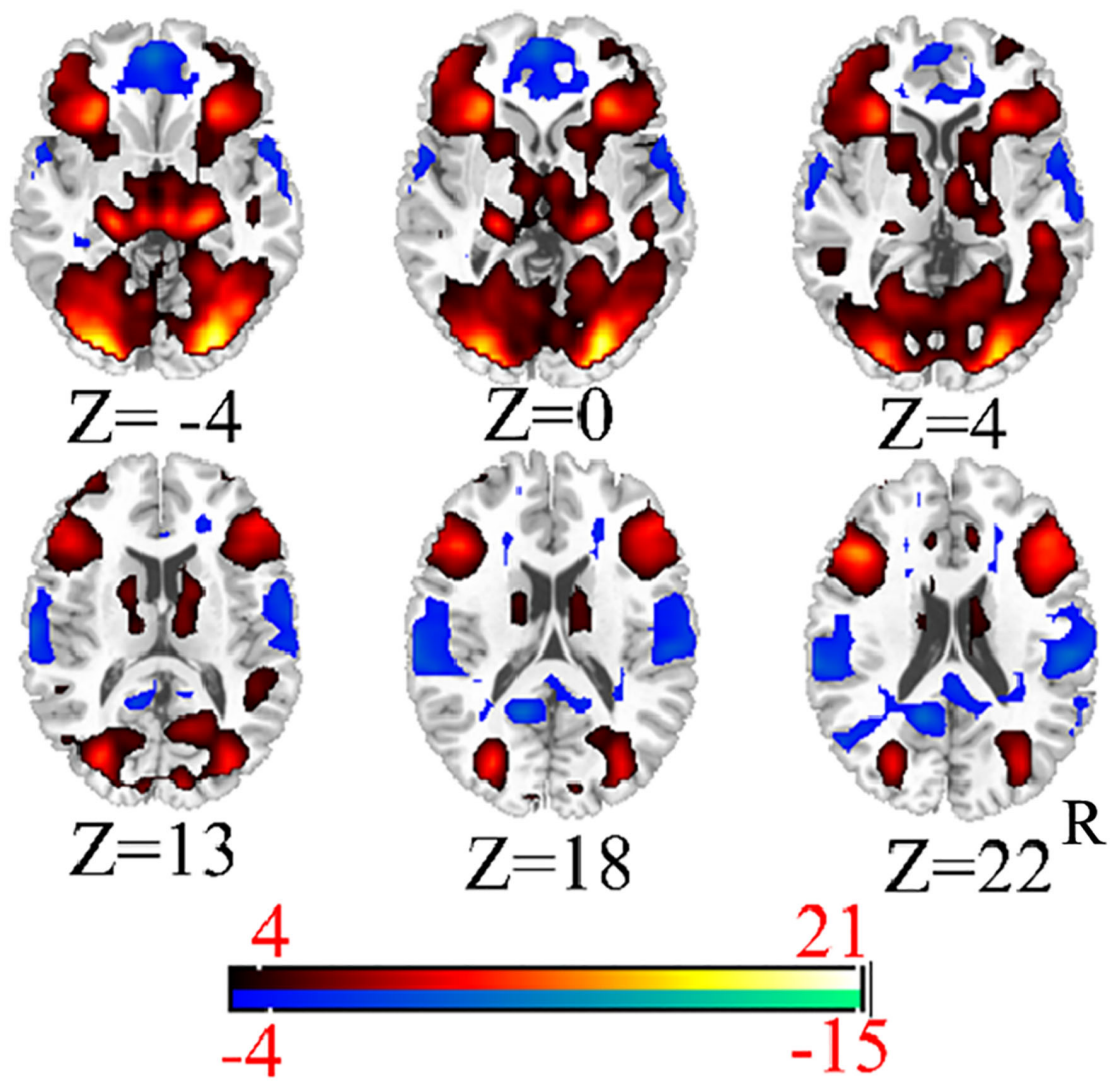
The functional activity mappings of emotional face.

First, we conducted a one-sample analysis of all the data in each condition to find more accurate activation regions. The results are shown in **Figures 3**, **4** (p < 0.001,cluster size > 50). Moreover, we analyzed the results of five single samples and extracted the coordinates of the regions with significant differences as the central coordinate point for our subsequent extraction of the regions of interest. Because we mainly explored the relationship between visual and high-level cognitive areas in the psychiatric risk group at work, we used tasks related to vision and emotion, and emotion is a high-level cognition, which conforms to the requirements of our research. Therefore, the brain regions we selected are mostly related to vision, emotion and cognition.

### 3.3 The results of RSA

The central coordinates of the main region of interest were the right inferior occipital gyrus, the right fusiform gyrus, the right triangular part of the inferior frontal gyrus, the left middle occipital gyrus, and the left middle temporal gyrus (**Table 2**). The results of RSA showed that under the 5hz and 20hz conditions, the low-risk group had stronger voxel activity similarity in the right inferior occipital gyrus and the right triangular inferior frontal gyrus than the high-risk group. Under the 5hz and 10hz conditions, the low-risk group also had higher voxel activity similarity in the right fusiform gyrus than the high-risk group. In terms of different tasks, the low-risk group also showed higher voxel activity similarity in the left middle occipital gyrus and left middle temporal gyrus in the 10hz and emotional picture response task states than the high-risk group. In general, the activity similarity of the same voxel in the same brain region in the low-risk group was higher than that in the high-risk group under different task conditions. Moreover, we conducted validation analyses using different ROI radii (4 mm and 9 mm) in addition to our original 6 mm ROI. The results remained consistent across these different ROI sizes, which supports the robustness of our findings.

**Table 2:** The coordinate of each ROI.

Region	MNI coordinates			Peak-t score	Cluster Size
	X	Y	Z		
Right middle temporal gyrus	39	-69	13	8.203	224
Right inferior occipital cortex	25	-84	-8	-5.063	73
Left inferior occipital cortex	-25	-84	-8	-7.875	63
Right Subfrontal gyrus triangularis	40	40	7	3.828	297
Left middle occipital gyrus	-36	-72	15	9.727	269
Right Subfrontal gyrus triangularis	46	23	22	13.13	428
Left Subfrontal gyrus triangularis	-47	-59	-2	5.891	60
Left lingual gyrus	17	-49	-2	-5.414	192
Right fusiform	30	-69	-8	9.515	236

## 4 Discussion

Schizophrenia is a complex disorder, and scientists are continually advancing their research on it, with new research methods emerging constantly. This progress offers hope for alleviating and potentially curing the disease. Research on the early stages of schizophrenia is particularly important for understanding its onset and progression. In RSA, the dependent measure is the similarity degree of voxel patterns under different experimental conditions. This method is more convenient and effective for examining the activity of voxels in relevant brain regions across time and stimuli, allowing for a clearer understanding of voxel activity patterns (20). Our results indicated that under 5Hz and 20Hz conditioned stimuli, there was strong representational similarity between two groups of subjects in the right inferior occipital gyrus and the right inferior frontal gyrus, with significant differences observed between the groups. Under 5Hz and 10Hz stimulation conditions, the similarity was stronger in the right fusiform gyrus region. This is attributed to similar voxel activity between the groups during the SSVEP experiment. Specifically, there was strong similarity in activity patterns in the left middle occipital gyrus and left middle temporal gyrus under the 10Hz condition and during the emotional face matching task. Across all the couplings identified, the high-risk group exhibited lower similarity compared to the low-risk group.

Previous studies often used fMRI to explore changes at the group level. However, a growing number of recent studies have begun to utilize magnetic resonance imaging to investigate individual differences (21, 22), introducing this method into the field of mental health research (23, 24). This approach is known as RSA (25–27). RSA falls within the framework of multivariate pattern analysis and examines the activity of many distributed neurons that function as mental representations. It offers researchers a richer method for revealing high-dimensional information in the structure of mental representations, as it relies on relative differences between voxels rather than within them. Using RSA to study the visual network of individuals at risk of mental disorders represents a bold and innovative attempt.

The human visual network is primarily composed of the WHAT and WHERE pathways. The WHAT pathway enables us to identify objects and recognize what we see, while the WHERE pathway helps us determine the specific location of these objects. The cooperation of both pathways allows for better identification and interaction with objects in our environment. The WHAT pathway begins in the occipital lobe, passes through the temporal lobe, thalamus, and insula, and finally reaches the frontal lobe, forming a connection from the primary visual cortex to higher cognitive areas (3, 28). Our results indicated that during the SSVEP task, the relevant brain regions of the WHAT pathway exhibited relatively strong representation similarity under different stimulus conditions, with the similarity in the low-risk group being higher than that in the high-risk group (29). This suggests that there is a reduction in the coupling of visually relevant areas in individuals at high risk under varying stimulus conditions.

Numerous fMRI studies have demonstrated that viewing emotional pictures increases cerebral blood flow (CBF) in limbic, frontoparietal, and higher-order visual structures compared to neutral images (30, 31). Our findings also revealed activation in these areas during the emotional face matching task. The emotional face recognition task engages not only visual processing but also analytical abilities and social functions. Social dysfunction is a core feature of schizophrenia and serves as a significant predictor of social functioning (32, 33). From a developmental perspective, the formation of the “social brain” is closely tied to experiences of emotional resonance and the development of emotional regulation (34). Our study identified a relatively strong coupling in the visual WHAT pathway through RSA, revealing that the high-risk group showed significantly lower coupling compared to the low-risk group, particularly in the middle occipital and middle temporal gyri. Under the 10 Hz stimulation condition and emotional picture stimuli, these regions were activated, demonstrating robust coupling between them. This suggests that they are fundamental areas within the visual network, responding to visual stimuli with high activation similarity and similar processing modes. Previous research indicates that the occipital and temporal lobes are primary cortical regions critical for visual processing. The significant differences observed between the high-risk and low-risk groups in these regions suggest that the high-risk group’s brain may have undergone changes affecting activation patterns and responses to visual stimuli. These differences could be attributed to subtle alterations in brain structure and functional connectivity arising from prolonged exposure to emotional distress or stress (35).

This study found that the high-risk group exhibited poorer representational similarity than the low-risk group under all stimulus conditions, and the coupling between related brain regions in the high-risk group was not as strong as in the low-risk group during various tasks. This was primarily observed in the right inferior occipital gyrus, right fusiform gyrus, right triangular part of the inferior frontal gyrus, left middle occipital gyrus, and left middle temporal gyrus. These regions are associated with the visual and emotion networks, and the abnormal coupling in these areas further indicates that the visual network has been compromised, which is consistent with previous studies.

## 5 Limitation

One key limitation of our study is the cross-sectional design, which limits our ability to draw causal inferences regarding whether the observed deficits serve as risk markers or are merely epiphenomena. Given the high heterogeneity among PRS subjects and the dynamic nature of their symptoms over time, future longitudinal follow-up studies are essential to better understand the dynamic progression of pathological features in this population. Additionally, our findings require further validation in larger, more homogeneous samples to more accurately elucidate the relationship between behavioral deficits and functional alterations.

## Data Availability

The original contributions presented in the study are included in the article/supplementary material. Further inquiries can be directed to the corresponding authors.
